# A graded tractographic parcellation of the temporal lobe

**DOI:** 10.1016/j.neuroimage.2017.04.016

**Published:** 2017-07-15

**Authors:** Claude J. Bajada, Rebecca L. Jackson, Hamied A. Haroon, Hojjatollah Azadbakht, Geoff J.M. Parker, Matthew A. Lambon Ralph, Lauren L. Cloutman

**Affiliations:** a Neuroscience and Aphasia Research Unit (NARU), School of Biological Sciences, The University of Manchester, UK; b Division of Neuroscience & Experimental Psychology, School of Biological Sciences, The University of Manchester, UK; c Division of Informatics, Imaging & Data Sciences, School of Health Sciences. The University of Manchester, Manchester, UK; d Bioxydyn Limited, Manchester, UK

**Keywords:** Spectral reordering, Connectivity based parcellation, Probabilistic tractography, Diffusion MRI, Temporal lobe

## Abstract

The temporal lobe has been implicated in multiple cognitive domains through lesion studies as well as cognitive neuroimaging research. There has been a recent increased interest in the structural and connective architecture that underlies these functions. However there has not yet been a comprehensive exploration of the patterns of connectivity that appear across the temporal lobe. This article uses a data driven, spectral reordering approach in order to understand the general axes of structural connectivity within the temporal lobe.

Two important findings emerge from the study. Firstly, the temporal lobe's overarching patterns of connectivity are organised along two key structural axes: medial to lateral and anteroventral to posterodorsal, mirroring findings in the functional literature. Secondly, the connective organisation of the temporal lobe is graded and transitional; this is reminiscent of the original work of 19^th^ Century neuroanatomists, who posited the existence of some regions which transitioned between one another in a graded fashion. While regions with unique connectivity exist, the boundaries between these are not always sharp. Instead there are zones of graded connectivity reflecting the influence and overlap of shared connectivity.

## Highlights

• A graded parcellation identified changes in connectivity across the temporal lobe
• Connective organisation of the temporal lobe was graded and transitional
• Two axes of organisation were found: medial-lateral and anterovental-posterodorsal
• While regions of distinct connectivity exist, their boundaries are not always sharp
• Zones of graded connectivity exist reflecting influence of shared connectivity

## Introduction

The temporal lobe is a complex region that supports multiple cognitive domains including language (Cloutman, 2013, Price, 2010), semantic processing (Lambon Ralph, 2014, Lambon Ralph et al., 2017), memory (Scoville and Milner, 1957), audition (Kaas and Hackett, 1999) and vision (Goodale and Milner, 1992, Grill-Spector and Malach, 2004). In order to understand its roles in these diverse cognitive functions, researchers have attempted to map the precise anatomical organisation within the temporal lobe, revealing an intricate functional architecture and regions of specialisation throughout the temporal cortex. For example, within the temporal lobe, antero-ventral and middle temporal areas have been found to be associated with semantic processing (Binder et al., 2009, Binney et al., 2010, Lambon Ralph et al., 2017), while medial areas have long been implicated in episodic memory (Scoville and Milner, 1957).

One way to understand the functional organisation of a region is to understand its structural composition. Traditionally, the exploration and mapping of structural/functional subdivisions within the cortex has been based primarily on cytoarchitecture but there has also been work on receptor distribution and other microarchitectural patterns. The laminar distribution of a given area, in conjunction with local microcircuitry and connectivity patterns determines its functional processing capabilities (c.f. Amunts and Zilles, 2015 for a full and in depth review). Indeed, the cortex does not exist as a detached entity and regions such as the temporal lobe are highly interconnected both locally and to other areas throughout the brain via white matter fibre bundles (Bajada et al., 2015, Catani,, 2007, Catani et al., 2012, Catani and Thiebaut de Schotten, 2008, Déjerine and Déjerine-Klumpke, 1895, Duffau, 2015). These structural connections are assumed to be a determinant of the functional capabilities of a cortical area, governing the nature and flow of information to and from an area, and can influence both its underlying neural architecture and its functioning (Anwander et al., 2006, Cloutman and Lambon Ralph, 2012, Johansen-Berg et al., 2004).

While there has been a lot of research mapping function to structure within the temporal lobe and reconstructing the white matter fibre bundles that course through it, there has been relatively little exploration of the organising principles underlying connective similarity in the temporal lobe. Parcellation schemes identify core regions within the target area of interest where there is high intra-regional similarity in relation to some aspect of their anatomical or functional anatomy, but comparatively low similarity with areas outside the sub region. From these parcellations, researchers are able to delineate key regions of anatomical distinction, and by inference, areas of functional specialisation (Cloutman and Lambon Ralph, 2013). However, there is evidence to suggest that such hard parcels may not always describe the true underlying nature of the data (Brodmann, 1909 pp. 120–122; von Bonin and Baily). Brodmann himself noted that “*not all these regions are demarcated from each other by sharp borders but may undergo gradual transitions as, for example, in the temporal and parietal regions.”* (Brodmann, 1909 p. 106).

In recent years with the advent of modern imaging techniques, researchers have begun to explore different ways to parcellate the cortex based on their patterns of connectivity described as connectivity-based parcellation (Eickhoff et al., 2015). Three main types of algorithms have been used. The first two are k-means clustering and hierarchical clustering (c.f. Eickhoff et al., 2015 for a full review). The third approach utilises principles of spectral graph theory to perform the parcellation (Cerliani et al., 2012, Devlin et al., 2006, Eickhoff et al., 2015, Haak et al., 2016, Johansen-Berg et al., 2004). The latter approach, often referred to as spectral reordering or a closely related Laplacian eigenmapping (Belkin and Niyogi, 2002, Cerliani et al., 2012, Johansen-Berg et al., 2004), allows for the investigation of the relationships between areas, whether these are graded or distinct, and hence is appropriate to investigate the organising principles of the temporal lobe.

Our aims were twofold. First, we wanted to establish whether the data supported graded regions within the temporal lobe. Second, we wanted to explore how connectivity similarity varied across the cortex.

In order to address these questions, the current study used spectral reordering, a data transformational technique, to explore the temporal cortex's connectivity. While not a clustering technique in the formal sense, the approach is well-established in the literature and its results have been validated (Anwander et al., 2006). We extended the method by projecting the reordered voxels into brain space. This allows one to elucidate the spatial pattern of connectivity across the cortex. We applied this technique to the temporal lobe and found that connectivity changes occur along a medial to lateral as well as anteroventral to posterodorsal axis. The tracts that underlie these axes were then explored. We finally discuss the possible functional processes that these gradations underpin. Throughout this paper we have referred to the current approach as a 'graded' parcellation. It is important to note that this is not to imply a presupposition about the underlying anatomical structure (indeed, the method allows both transitional and more discrete boundaries to emerge), but to differentiate it from more traditional methods which impose the delineation of hard boundaries.

## Materials and methods

### Image acquisition

A dataset containing structural (T1 and T2-weighted), and diffusion-weighted MR images from 24 healthy participants (mean age 25.9 years, range 19–47 years; 11 females) was used. All participants were right handed, as determined by the Edinburgh Handedness Inventory (Oldfield, 1971). The study was approved by the local ethics committee and all participants gave their informed consent. The images were acquired on a 3 T Philips Achieva scanner (Philips Healthcare, Best, The Netherlands), using an 8 element SENSE head coil. Diffusion-weighted images were acquired with a pulsed gradient spin echo echo-planar sequence with TE=59 ms, TR ≈ 11884 ms (cardiac gated using a peripheral pulse monitor on the participant's index finger (n=21), or using electrocardiography (n=3)), Gmax=62 mT/m, half scan factor=0.679, 112×112 image matrix reconstructed to 128×128 using zero padding, reconstructed in-plane voxel resolution 1.875×1.875 mm^2^, slice thickness 2.1 mm, 60 contiguous slices, 61 non-collinear diffusion sensitization directions at b=1200 s/mm^2^ (Δ=29. ms, δ=13.1 ms), 1 at b=0, SENSE acceleration factor=2.5. In order to correct susceptibility-related image distortions, two volumes were obtained for each diffusion gradient direction with inversed phase encode directions, with distortion correction carried out using the method described in Embleton et al. (2010). In order to obtain a qualitative indication of distortion correction accuracy, a co-localized T2-weighted turbo spin echo scan (in-plane voxel resolution of 0.94×0.94 mm^2^, slice thickness 2.1 mm) was obtained. A high resolution structural T1-weighted 3D turbo field echo inversion recovery scan (TR ≈ 2000 ms, TE=3.9 ms, TI=1150 ms, flip angle 8°, 256×205 image matrix reconstructed to 256×256, reconstructed in-plane voxel resolution 0.938×0.938 mm, slice thickness 0.9 mm, 160 slices, SENSE factor=2.5), was acquired in order to obtain high accuracy anatomical data on individual subjects which were used to define individualised anatomical seed regions

### Tractography

A temporal lobe region of interest was created which included all voxels within the temporal lobe at the boundary between the grey matter and the white matter (Anwander et al., 2006). To do this, each participant's skull stripped (FSL BET; Smith, 2002) T1-weighted image was co-registered to the distortion-corrected diffusion images using FSL's linear affine transformation (FLIRT) (Jenkinson et al., 2002, Jenkinson and Smith, 2001). The interface between the grey and white matter of the co-registered T1 image was then obtained using FSL's FAST algorithm to obtain a partial volume map of white matter, which was binarised with no threshold to ensure that the map overlapped the edge of the grey matter – the grey matter to white matter interface (GWI). The perimeter voxels of this map (the GWI) were extracted using an in-house MATLAB script. The GWI was then masked to include only those voxels within the temporal lobe. A temporal mask was first defined in MNI space using the MNI structural atlas within FSL (Collins et al., 1995, Mazziotta et al., 2001). This mask was then normalised and co-registered to each participant's native diffusion space. In order to ensure full temporal lobe coverage, the original probabilistic temporal mask was leniently thresholded. This resulted in the region of interest encroaching (or ‘bleed’) into other lobes (for example the frontal lobe across the sylvian fissure). In order to ensure that only the temporal lobe was used as a region of interest, the masks were all manually reviewed in native space. The corrected temporal mask was then used to mask the GWI to create the temporal GWI seed regions of interest used for tracking.

Unconstrained probabilistic tractography was performed from every individual voxel in the temporal lobe GWI using the probabilistic index of connectivity (PICo) algorithm (Parker and Alexander, 2005, Parker et al., 2003), which sampled the voxel-wise diffusion probability distribution functions (PDFs) generated via the constrained spherical deconvolution (Tournier et al., 2007) and model-based residual bootstrapping method (Haroon et al., 2009a, Haroon et al., 2009b, Jeurissen et al., 2011). During tracking, 10,000 streamlines were propagated from each seed voxel, with step size for streamline propagation set to 0.5 mm. An exclusion mask was created and used to avoid path propagation through the grey matter and tracts anomalously jumping sulcal boundaries and gyri. The streamlines were set to stop if they hit the exclusion mask, if the path length of the streamline was greater than 500 mm, or if the curvature of the streamline was greater than 180°. For each individual seed voxel within the temporal lobe GWI (approx. 3000), the number of streamlines originating from the seed which reached a given voxel in the brain was recorded, generating a tractographic connectivity profile for each temporal GWI seed voxel.

### Spectral reordering and graded parcellation

**Fig. 1:**
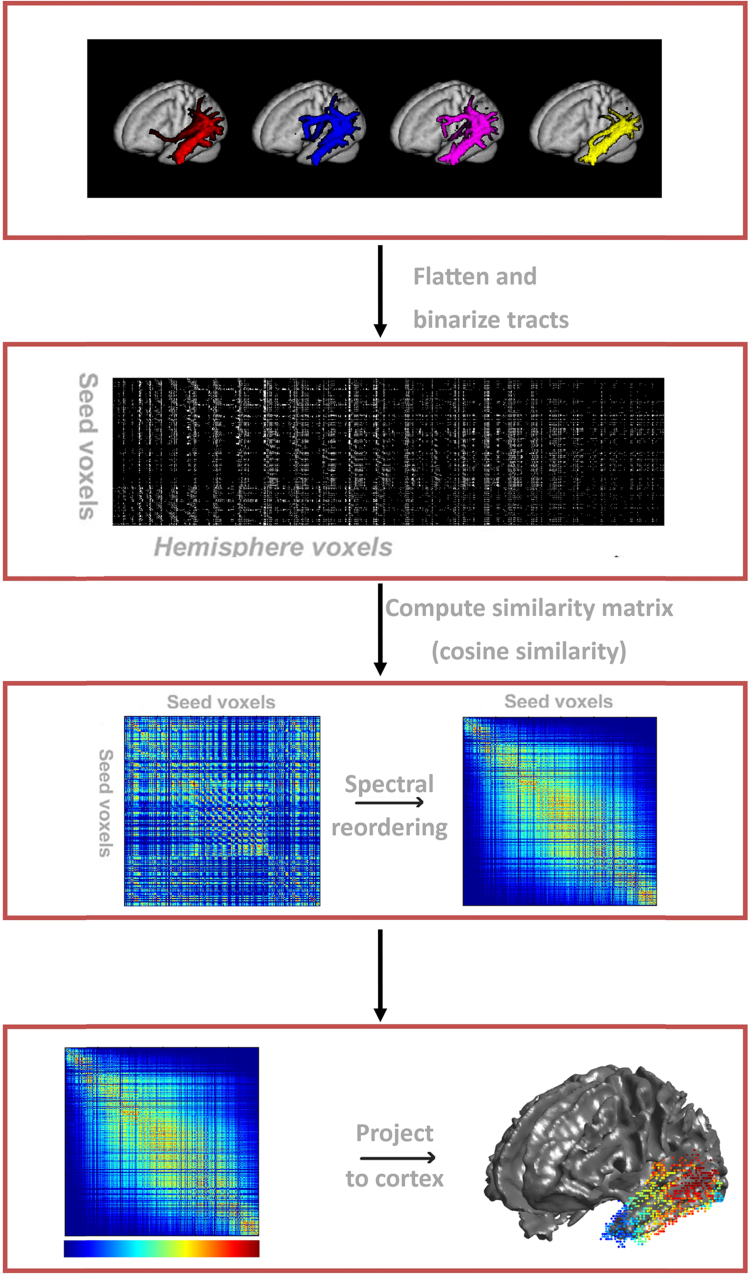
–The pipeline to create a graded parcellation of an individual temporal lobe. a) Sample of connectivity profiles from adjacent temporal seed voxels. b) Connectivity profiles are flattened and concatenated to one another to create a 2D matrix representing connectivity of seeds voxels to other voxels. c) A pairwise similarity algorithm is run on b) to generate a similarity matrix. The similarity matrix is spectrally reordered such that seed voxels with strong similarity between their patterns of connectivity are positioned together. d) The reordered matrix is projected onto the brain to visualise the resulting graded parcellation.

The graded parcellation via spectral reordering was carried out based on the work by Johansen-Berg et al. (2004) as follows (for a pipeline, see Fig. 1). The tractographic connectivity profiles of each individual participant's temporal GWI seed voxels were first normalised to a common group space using SPM's DARTEL (Ashburner, 2007). Each seed's connectivity profile was then thresholded at 0.05 percent of the maximum in order to remove noise, in keeping with a similar study by Devlin et al. (2006). The resulting 3D tractographic volumes for each seed voxel (Fig. 1a) were downsampled by a factor of 2 due to the machine's memory constraints. The image was binarised and flattened into 1*×m* row vectors where the columns (*m*) represented every point in the brain (Johansen-Berg et al., 2004). The participant tractographic connectivity profiles in row vector form were concatenated into individual *n×m* matrices, where each row (*n*) represented the connectivity profile of an individual temporal seed voxel with every other voxel in the brain (*m*) (Fig. 1b). If a column contained all zero entries, it was removed (creating an *n×m’* matrix) to further reduce memory load. In order to determine which temporal lobe seed voxels shared similar connectivity profiles, a pairwise similarity algorithm was run on the temporal connectivity matrix to calculate the cosine of the angle between each pair of rows in the above matrix. This generated an *n×n* symmetric matrix with all of the temporal seeds plotted against each other, and values representing the degree of similarity between each pair of seed voxels in their patterns of tractographic connectivity.

The spectral reordering algorithm was then applied to the matrix, permuting it and forcing seed voxels with strong similarity to be positioned close together (Fig. 1c) (see supplementary text for a MATLAB^®^ implementation, see Barnard et al. (1995) and the supplementary materials from Johansen-Berg et al. (2004) for further details on the mathematical background of the algorithm). To obtain the final graded parcellation, the temporal seed voxels in the reordered matrix were projected back onto the brain, enabling reordered voxels within the similarity matrix to be visualised in reference to their anatomical location. To visually code the position of each seed voxel within the matrix a graded colour spectrum was used. This colour spectrum transitioned across the matrix from left to right such that, for example, those voxels which grouped together on the far left of the matrix were coloured blue and those clustered together on the far right coloured red (Fig. 1d).

#### Group analysis

To perform the graded parcellation at the group level, each individual's tractographic connectivity profiles were mapped onto a group template GWI. The group template GWI was created using the steps for creating the individual participant temporal lobe GWI as described above, but with the group averaged template brain produced by SPM's DARTEL. Each voxel in every participant's temporal GWI was then mapped onto its nearest neighbour (in Euclidean distance) onto the group template. Since all individual GWIs had less voxels (approx. 3000) than the template GWI (approx. 5000), some individual voxels were mapped onto more than one voxel on the template GWI. Once the mapping was complete, each voxel on the template GWI had 24 binarised tracts associated with it (one for each participant). These were averaged to produce a probabilistic tract across the 24 individuals. These tracts were then flattened to produce an *n×m* matrix, where each row (*n*) represented the connectivity profile of an individual temporal seed voxel with every other voxel in the brain (*m*) in the same way that the individuals’ data were processed. Every entry in this matrix, however, ranged from zero to one where zero denoted that no individual had a binarised tract visit that voxel and one denoted that all individuals had a binarised tract visit that voxel. The remainder of the procedure was carried out in the same manner as for the individual participant analyses.

#### Graded connectivity analysis

To determine whether the temporal cortex was characterised by gradations or whether clear cortical groupings were sufficient to describe the connectivity pattern, two methods were used. First, a qualitative visual inspection of the reordered matrix was performed to identify patterns and gradients within the spectrally reordered connectivity profiles. Second, a quantitative measure of gradation was employed which used information provided by the second smallest eigenvalue (λ_2_) of the Laplacian. This eigenvalue will be close to zero if the similarity matrix contains groups of voxels which are strongly interrelated within but not between groups (i.e., defined groups with weak gradation between them), while higher numbers are associated with greater levels of gradation between areas.

**Fig. 2:**
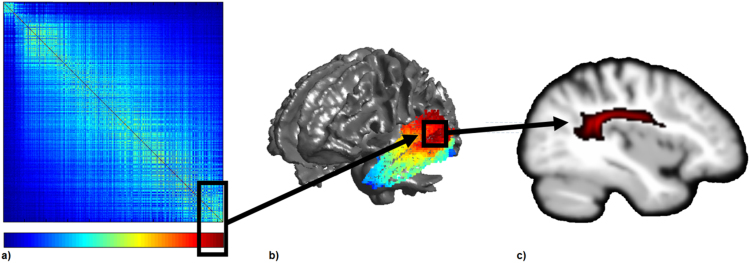
An illustration of how the underlying connectivity of the graded parcellation can be explored. The black boxes indicate corresponding region in the matrix (a) and their approximate location on the cortex (b). The colour bar under the matrix represents the mapping of colour from the matrix to the cortex. This example shows the far right hand corner of the matrix represented at the red end of the colour bar, which maps onto the posterior superior and middle temporal gyri. A sagittal slice through the underlying tract(s) from a voxel in this region is displayed in panel c.

In order to further explore the relationships between the graded parcellation and the underlying structural connectivity, tractographic connectivity profiles associated with different parts of the matrix were visualised. Each seed voxel in the matrix is associated with both an area of cortex and its underlying connectivity profile (see Fig. 2). As such, areas of the matrix that correspond to particular cortical regions of interest can be identified (or vice versa), and the connectivity profiles in those specific areas extracted, summed and visualised. In the current study, in line with the four main tracts of the temporal lobe, four tractographic connectivity profiles associated with different parts of the matrix were visualised.

### Validation and consistency

Two approaches for assessing the across-participant consistency were conducted. The first approach sought to assess how each individual participant's spectral reordering correlated with the group voxel orderings, using a leave-one-out cross-validation approach. To do this, a group dataset was created which included the connectivity profile data of every participant excluding one (the test participant). A permutation vector was obtained for this group-minus-one dataset using the group analysis method described above, which defined how the group-minus-one data should be spectrally reordered. This was taken to be the predicted ordering for the individual test participant. The test participant's data were then permuted for a first time according to the predicted permutation vector, which reordered the participant's data to the group-minus-one predicted order. This dataset was then reordered using the spectral reordering algorithm to obtain the test participant's individual ordering. This actual ordering was then correlated with the predicted ordering to provide a measure of consistency. This procedure was repeated for every participant.

The second approach assessed which voxels showed the greatest variability across participants, in order to assess which areas of the ordering are most reliable. To do this, the group ordering for the complete group of participants (n=24) was taken as the reference ordering. Each participant's data was then first permuted to the reference ordering and then spectrally reordered to obtain the actual ordering for each participant. The absolute deviation of each voxel in a participant's ordering to that in the reference order was then computed. Finally, for each voxel, the mean absolute deviation across participants was calculated and plotted onto the brain (see Fig. 5).

## Results

**Fig. 3:**
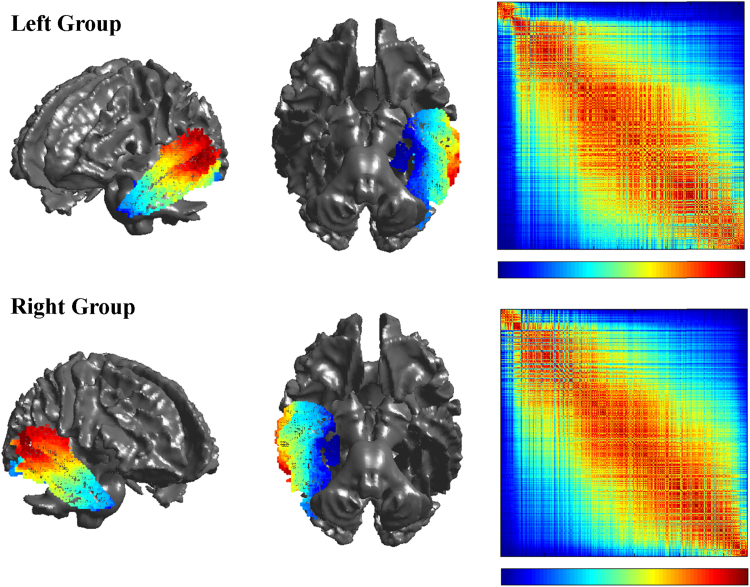
– Group level graded parcellation results of the left and right temporal lobes, displaying lateral and ventral views projected onto the brain as well as the spectrally reordered matrices on which they are based. The colour bar below the matrices has two representations: 1) the level of similarity between left (blue) – right (red), 2) a mapping between the seed in matrix space and in brain space (for example the left most seed (column) in the matrix is coloured blue on the brain while the right most seed is coloured red).

The results from the group level graded parcellation for both the left and right hemispheres are presented in Fig. 3. An examination of the individual level parcellations for each participant revealed a similar pattern of connectivity to that of the group level reordering across all 24 participants (see Supplementary Materials). An examination of the parcellations reveals that the structural connectivity of the temporal lobe is arranged along two main axes of organisation, one medial to lateral and the other from the anteroventral to posterodorsal temporal lobe. This organisational structure was observed for both the left and right hemispheres, which demonstrated very similar parcellation results (Fig. 3).

**Fig. 4:**
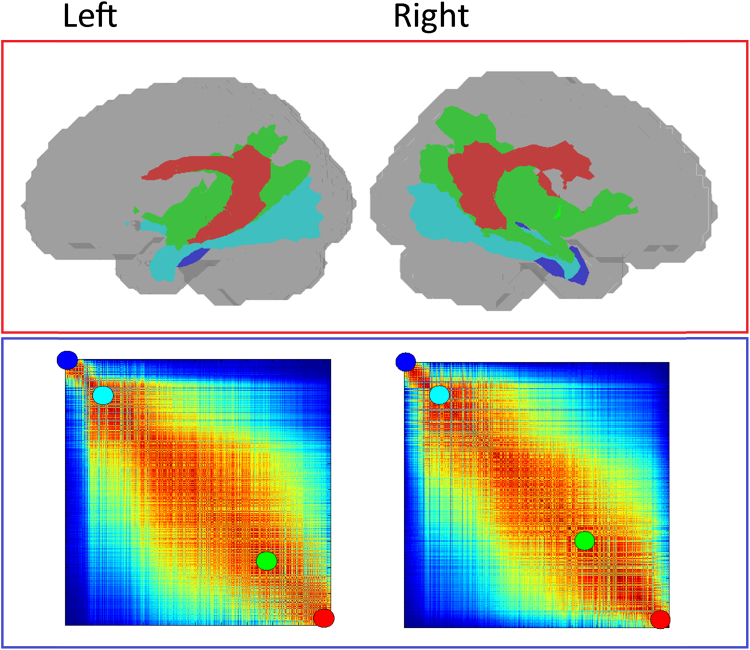
Sample connectivity profiles underlying the group reordering for the left and right hemispheres. Four group connectivity profiles were selected at the extremes of the matrix and within the graded section to depict the general order of connectivity profiles. The left and right hemisphere show a similar trend of fibres where early in the ordering the cingulum is prominent followed by more intra-lobar and temporo-frontal tracts. The other end of the matrix is dominated by the temporo-parietal middle longitudinal fasciculus and the arcuate fasciculus.

In addition to the two principal axes of organisation, further examination of the sorted matrix and its corresponding projection onto the cortex reveals areas of differentiation throughout the temporal lobe. The main cortical areas at the extreme ends of the matrix are associated with relatively well-defined and distinct core subregions. These two prominent subregions are located around the posterior superior and middle temporal gyrus (red areas in Fig. 3), and in the parahippocampal gyrus (blue areas). To explore the white matter architecture underlying these strong divisions in cortical connectivity, the connectivity profiles of voxels corresponding to the two regions were visualised (Fig. 4; files are available in Supplementary Materials). An examination of these underlying connections reveals distinct tracts which uniquely dominate the connectivity of the two core subregions, with the most ventromedial (blue, Fig. 4) temporal regions dominated by connections coursing through the parahippocampal branch of the cingulum bundle, and the most posterodorsal (red, Fig. 4) temporal regions associated primarily with connections dominated by the arcuate fasciculus.

In contrast to these two most differentiated regions, the remaining areas of the temporal cortex exhibit a pattern of more graded and transitional connective subdivisions. This is supported by examination of the second smallest eigenvalue, which was not close to zero in either hemisphere (λ_2_ = 0.63 in the left hemisphere and λ_2_ = 0.64 in the right), indicating that the groupings were not well differentiated, and there were similarities between voxels across groups. This, along with a visual inspection of the matrix, points to a graded transition between the connective subregions. Looking at the transitions across the parcellation matrix, it can be seen that the ventral surface of the temporal cortex involving the more lateral anterior aspects of the temporal lobe along the fusiform gyrus and parts of the inferior temporal gyrus (blue to cyan areas in Fig. 3), demonstrates a pattern of graded yet spatially contiguous connectivity profiles. An examination of the underlying connective tracts within this region shows connections predominantly via the inferior longitudinal fasciculus (cyan tracts in Fig. 4).

Moving further along the matrix, there appears to be a movement away from the more ventral regions, with the anterior superior temporal gyrus (orange and yellow areas in Fig. 3) demonstrating a connectivity profile more similar to that of posterior superior and middle temporal areas. An examination of the underlying tracts in Fig. 3 reveals that this area is connected via the middle longitudinal fasciculus (Green), a tract which runs along the anterior-posterior course of the superior temporal gyrus, and which overlaps with the arcuate fasciculus at its more posterior end (Makris et al., 2009).

In an area of the matrix intermediary to these ventral and posterodorsal regions, there is a region involving the middle temporal gyrus/sulcus (cyan to green areas in Fig. 3), which is associated with a mixed transitional profile, demonstrating connectivity similar to both the ventral surface and the anterior superior temporal gyrus. This transitional zone appears to be associated with fibre tracts which strongly overlap anatomically with the origin/termination areas of both the inferior and middle longitudinal fasciculi, driving the similarity between the connectivity profiles of these temporal subregions.

### Validation and consistency

The individual's orderings all highly correlated with their predicted orderings (spearman rho range: 0.84–0.96). All correlations were significant (p<0.001). This implies that the group average (minus the individual to be predicted) connective gradations predict the individual gradations very well.

**Fig. 5:**
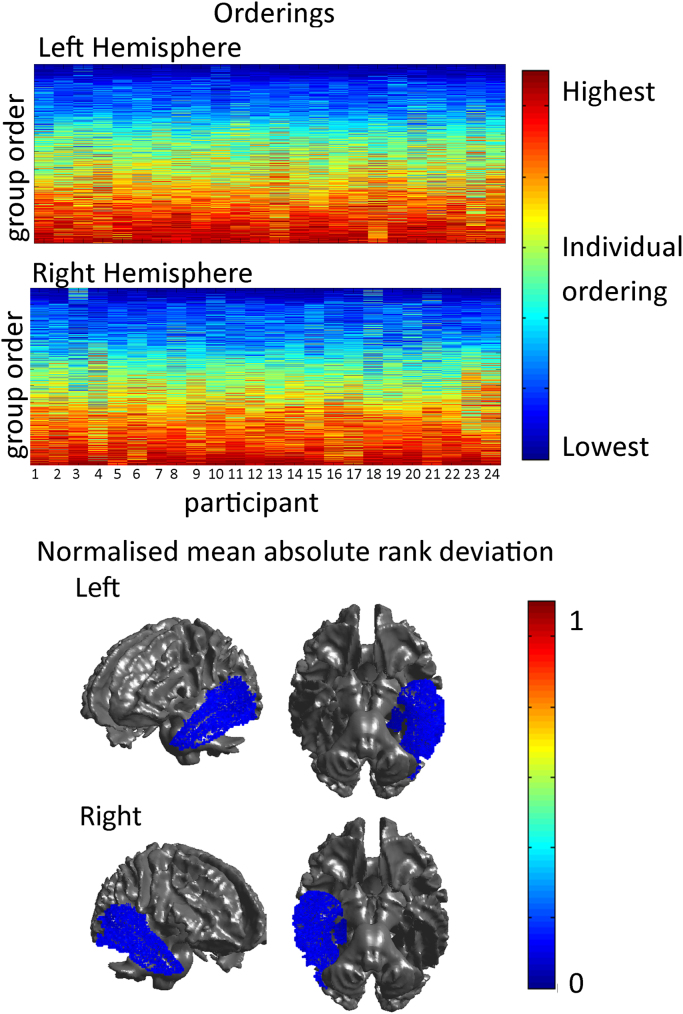
Upper Panel: The individual orderings across all the individual 24 participants. The colours represent the individual's ordering while the y-axis represents the group ordering. Lower Panel: the normalised mean absolute rank deviation for each voxel plotted on the cortex. All voxels have very low mean absolute rank deviations with the lowest being in the medial temporal lobe and the dorsolateral temporal lobe.

All voxels were highly consistent in their ordering with the most consistent voxels being in the medial temporal lobe and the dorso-lateral temporal lobes (see Fig. 5). This is expected as they are the most separable connectivity profiles. Therefore they will be consistently identified as distinct from other regions of the temporal lobe.

## Discussion

The current study utilised a data-driven technique to produce a graded parcellation of temporal lobe based on its varying patterns of underlying structural connectivity. The parcellation approach allowed for the exploration of not only regions of distinct connectivity (as traditionally identified via hard parcellation methods), but also the relationship between these subregions across the temporal lobe as a whole. The results revealed two key organisational principles underlying the connective architecture of this structurally and functionally complex region.

First, the overarching patterns of connectivity across the temporal lobe are organised along two key structural axes. The first axis is characterised by a primary medial to lateral change of connectivity across the temporal lobe, driven primarily by the connective dominance of the cingulum bundle at the medial extreme and the arcuate fasciculus at the dorsolateral end (Catani et al., 2005, Catani and Mesulam, 2008). The second axis arose along an anteroventral to posterodorsal orientation, which reflected the dominance of connections via the inferior longitudinal fasciculus and temporo-frontal fibres for anterior ventral regions, and the arcuate fasciculus at the most posterior and dorsal end of the temporal lobe.

The second key principle governing the connective organisation of the temporal lobe is the overall graded and transitional nature of the patterns and changes in connectivity. Early neuroanatomists attempting to delineate patterns of temporal (and other brain) subdivisions based on cytoarchitecture, found that while dissociable regions do exist, other boundaries are not clearly delimited but instead were characterised by a pattern of blending between neighbouring regions (c.f. Brodmann, 1909 pp. 120–122; von Bonin and Baily, 1961; see Amunts and Zilles, 2015, for a review). Recent work has also focused on the importance of gradients within both structural and functional neural architecture (Cerliani et al., 2012, Haak et al., 2016). The current study reiterates these findings with respect to regions’ connective architecture. We find that the temporal cortex is comprised of a number of core regions underpinned by unique and dissociable structural connectivity, each associated with specific underlying fibre tracts. However, the boundaries between these subregions are not sharp, and instead demonstrate transitional zones of graded similarity reflecting the influence and overlap of shared connective pathways. In the current study, two distinct core regions were identified, one involving the posterior superior and middle temporal gyri and the other the parahippocampal gyrus. These two regions reflected the most connectively disparate areas within the temporal lobe, and correspondingly were associated with disparate and non-overlapping fibre tracts, namely the arcuate fasciculus and the cingulum bundle. Transitioning between these two extremes, are subregions involving the ventral temporal surface (including the fusiform and inferior temporal gyri), middle temporal gyral/sulcal areas, and the anterior superior temporal gyrus. While these less distinct subregions are also associated with differential white matter fibre bundles (namely, the inferior longitudinal fasciculus, the temporo-frontal/inferior fronto-occipital fasciculus, and the middle longitudinal fasciculus respectively), they appear to comprise less spatially distinct pathways with strongly overlapping origin/termination areas. Indeed, the close similarity with neighbouring parahippocampal ventral areas may reflect the existence of abundant U-shaped fibres that connect adjacent areas of cortex within these ventral temporal regions (Catani et al., 2003, Davis, 1921).This is consistent with the finding that individual voxels in the brain may be connected to distant areas by more than one fibre bundle (Déjerine and Déjerine-Klumpke, 1895, Krestel et al., 2013). The difference between the distinct core subregions and these transitional areas may also reflect the relative dominance of inter- versus intra-regional connections, with those areas demonstrating more spatially-contiguous graded connectivity profiles found to demonstrate high within-lobe short-range temporal interconnectivity (Binney et al., 2012).

### The connectivity axes and their putative functional significance

**Fig. 6:**
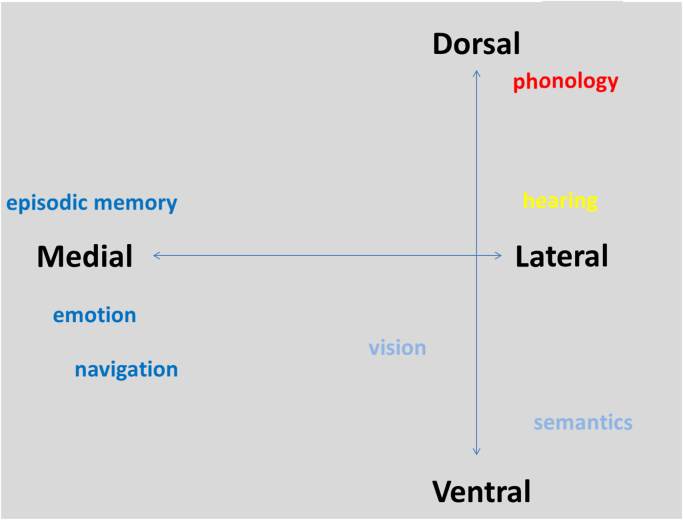
A schematic showing the structural axes of connectivity and putative cognitive functions that map onto these axes.

The current results elucidated the principal axes in which changes in connectivity across the temporal lobe occurred (see Fig. 6). Importantly, while other connectivity-based parcellation approaches may indicate different subregions in the medial, ventral and dorsal temporal lobe, the current approach was able to provide additional information about the structural relationships between these regions.

The axes of structural organisation identified in the current study can be seen to mirror major functional subdivisions found within the temporal lobe. The medial to lateral shift in connectivity has strong correspondences in the functional divide between medial temporal lobe's episodic memory, emotion and spatial navigation functions (Burgess et al., 2002, Dolcos et al., 2004, Guderian et al., 2009, Park and Rugg, 2010, Rugg et al., 2002), and the diverse lateral temporal lobe's functions including linguistic, semantic, auditory and visual processing (Goodale and Milner, 1992, Grill-Spector and Malach, 2004, Hickok and Poeppel, 2004, Kaas and Hackett, 1999, Kaas and Hackett, 2000, Saur et al., 2008). Episodic memory has long been associated with core regions within the medial temporal lobe and related connected areas, including the hippocampal and parahippocampal gyri, and the posterior cingulate gyrus (Park and Rugg, 2010, Rugg et al., 2002, Scoville and Milner, 1957). These regions share strong connections through the cingulum bundle, which has been shown to be associated with episodic memory performance and impairment (Lockhart et al., 2012, Metzler-Baddeley et al., 2011).

In contrast, the lateral and ventral temporal cortex has been associated with a wide range of cognitive tasks. In the left hemisphere, language functioning within the temporal cortex has been found to be more distributed but with a strong dominance of more lateral structures including Heschl's gyrus, and the superior, middle, and inferior temporal gyri (particularly their posterior sections) with key connectivity via the arcuate fasciculus; (Axer et al., 2013; Catani et al., 2005)). Bilaterally, it also underpins audition, vision and semantic processing (Goodale and Milner, 1992, Kaas and Hackett, 2000, Lambon Ralph, 2014, Lambon Ralph et al., 2009). The medial-lateral shift found in the current study appears to map onto the anatomical and connective patterns found between memory and other cognitive functions within the temporal lobe.

The anteroventral-posterodorsal axis of connectivity found predominantly along the lateral surface also seems to correspond to key functional subdivisions within the temporal lobe. In the left hemisphere, it most clearly mirrors the division between phonological and semantic processing, or the dorsal and ventral ‘language’ pathways (Cloutman, 2013, Hickok and Poeppel, 2004, Parker et al., 2005, Saur et al., 2008, Saur et al., 2010). While phonological processing within the temporal lobe has been found to be located within the more dorsal and posterior regions, particularly those connected to the dorsal language pathway via the arcuate fasciculus, (lexical) semantic processing has been particularly associated with more anterior and ventral areas including the temporal pole, anterior fusiform and inferior temporal gyri, commonly implicated within the ventral ‘language’ pathway (Axer et al., 2013, Duffau et al., 2013, Ueno et al., 2011). As such, the principal axis of organisation along the lateral surface may reflect connectivity changes associated with the relative functional specialisation of the dorsal and ventral pathways. In support of this, studies have found that the middle longitudinal fasciculus, particularly associated with the mid anterior temporal subregion in the current parcellation, is implicated in both semantic and phonological processing networks (Saur et al., 2010).

A similar pattern of graded connectivity was also observed for the right hemisphere, where it would seem less likely that such divisions were associated with linguistic functioning. However, it is important to note that in models of dorsal-ventral stream language processing, the ventral (temporal) pathway is much more bilaterally organised than the dorsal pathway (Hickok and Poeppel, 2004). In contrast, the dorsal pathway in the right hemisphere is more commonly associated with visuospatial processing, predominantly involving fronto-parietal areas, but with some evidence of a role of posterior temporal regions, particularly those around the temporoparietal junction (Bartolomeo et al., 2007, Chechlacz et al., 2013). As such, the axis of organisation along the lateral surface seen in the right hemisphere may reflect the division between ventral (lexico-)semantic processes and dorsal spatial processes. Additionally, in relation to linguistic functioning in the right hemisphere, the processing of speech prosody has generally been found to be right-lateralised, involving posterior temporal areas including the superior and middle temporal gyri (Merrill et al., 2012, Mitchell et al., 2003, Sammler et al., 2015). Interestingly, there is also evidence that this prosody network may be organised along a dorsal-ventral division, with an auditory-ventral pathway along the superior temporal lobe, and an auditory-motor dorsal pathway involving posterior temporal and inferior frontal/premotor areas (Sammler et al., 2015).

A final finding of note within the current study was the observation that unlike the more phonological- and memory-based temporal regions, those implicated in semantic processing were associated with high levels of graded connectivity between the areas involved. Previous proposals have suggested that the anterior temporal lobe plays a crucial role in conceptual representations (Lambon Ralph et al., 2010, Patterson et al., 2007). More recent functional imaging and connectivity studies have found that the anterior temporal function may be more graded in nature with partial specialisations arising from the differential patterns of connectivity (Binney et al., 2012, Visser et al., 2012, Visser and Lambon Ralph, 2011). Thus, the further ventro-anteriorly along the temporal lobe processing moves, the less specific the areas become to a particular sensory modality, instead becoming increasingly transmodal (Lambon Ralph, 2014, Rice et al., 2015, Visser et al., 2010). The transitional gradations along the anterior-posterior extent of the temporal cortex observed in the current connectivity parcellation study are consistent with these graded shifts and convergence of information along the temporal lobe (Binney et al., 2012, Lambon Ralph et al., 2017).

### Methodological considerations

Both dissection and tractography suffer from a heavy dependence on the researcher's anatomical knowledge in order to gain meaningful results. The approach used in this paper draws its strength from being data-driven and hence, has very little reliance on the user's potential prior biases.

#### Tractography

A common criticism of tractography is that it generates many false positive as well as false negative connections (Azadbakht et al., 2015). While this is true for all tractography studies, the impact of this limitation is mitigated in tractographic parcellation since accurate tracing is not essential to look for similarities and differences in a particular voxel's tractographic fingerprint (Johansen-Berg et al., 2004). Clearly, errors in the quality of the underlying data can influence the parcellation profile but in a potentially less dramatic way than false positive and negative tracts affect traditional tractography experiments. Related to this, it could also be argued that the gradations shown in the current study are simply an artefact of the imprecision of tractography and an inability of the method to demarcate clear boundaries. While it is possible that a degree of the gradation found may be due to error in the tracking process, we believe this error is highly unlikely to be the sole cause of the graded boundaries observed since the results match with well-known fibre bundles and functional subregions. Histological studies of fibre pathway terminations have observed patterns of interdigitating termination points for many regions throughout the brain (Selemon and Goldman-Rakic, 1985), which, alongside the cytoarchitectonic evidence from Brodmann and his contemporaries, suggest that the graded nature of the areal boundaries identified in the current study underlie a fundamental organisational principle of cortical architecture.

#### Spectral reordering and graded parcellation

Connectivity based cortical parcellations have, to date, focused primarily on the delineation and segmentation of clear and distinct independent regions, that is, hard parcellations (Anwander et al., 2006, Johansen-Berg et al., 2004, Thiebaut de Schotten et al., 2014). However, from the early days of parcellation it was understood that while some areas of the brain were in fact distinct regions, other zones showed more graded differences to one another (Brodmann, 1909). Indeed, more recent studies have provided additional support for the potential graded nature of anatomical boundaries, finding cytoarchitectural and connective gradations in areas such as the insula (Cerliani et al., 2012, Cloutman et al., 2012, Mesulam and Mufson, 1982). The results of the current study also emphasise this finding, with an examination of the second smallest eigenvalue (which should be close to zero if the graph is well clustered), indicating that the current connectivity data did not form well-defined and well-clustered elements. This is not to negate the idea that sharp boundaries do exist within the brain, or that gradation may be the only way in which the assumption of distinct anatomical homogeneity between parcellated regions may be violated. Heterogeneity may occur within a region when defined by one anatomical architecture or method (e.g., cyto-, myelo-, histochemical) but not others. Additionally, interdigiation of connections rather than gradation is known to occur in some relatively structurally homologous regions, such as motor-cortical projections within dorsal striatal sites (Shepherd, 2013). What the current results regarding the connectivity of the temporal lobe stress is that the strong divisions between cortical areas delineated by classic parcellation approaches may not fully reflect the true underlying nature of the cortex, and new approaches which enable these important architectural characteristics to be revealed are needed. The current study implemented such an approach using spectral reordering.

There have been several papers in the recent literature that have investigated different methods of connectivity-based parcellation of the cortex (see Cloutman and Lambon Ralph, 2012; Eickhoff et al., 2015; Klein et al., 2009 for comprehensive reviews). Spectral reordering was first introduced by Johansen-Berg et al. (2004). It is a technique derived from spectral graph theory that uses the Fiedler vector (Fiedler, 1973) to reorder the data at hand in such a way that points that are similar to one another are forced together within the ordering. A focus on reordering has the advantage of being able to probe a dataset without first assuming that, the data fall into neat clusters and second, without the need for determining an *a priori* number of clusters. This may be an advantage when not enough knowledge is known about the underlying architecture of a given region to inform clustering approaches, when examining a region where there is no obvious number of clusters that the cortex can be parcellated into, or when the region displays gradations in connectivity. Despite these advantages, spectral reordering may only show the most predominant connectivity gradients (or axes of connectivity) in the cortex and may miss finer-grained details in localised zones. It is hence important to state that one must not over-interpret the details of the parcellation but focus primarily on the overall pattern of connectivity it produces. Additionally, the technique may not be able to delineate some architectural structures, and may fail to elucidate some complex regional organisations such as interdigitated islets embedded within a region. It is also important to note the data reduction limitations of the approach that visualises a three dimensional structure in only one dimension. However, despite its limitations, spectral reordering is a useful technique to elucidate the main gradations in structural connectivity of an area. There is clear possibility for additional future work, such as the embedding of the graph into a three dimensional plane and visualisation of more finessed gradations found.

## Conclusions

This paper explored an approach to extract the major cortical gradient in the temporal lobe based on its patterns of structural connectivity. Two key results have been described in this paper. First, the connective organisation of the temporal lobe is graded and transitional. While core regions with unique connectivity exist, the boundaries between these sub-regions may not always be sharp. They demonstrate zones of graded connectivity reflecting the influence and overlap of shared connective pathways. Second, the overarching patterns of connectivity across the temporal lobe are organised along two key structural axes: medial to lateral as well as anteroventral to posterodorsal. The structural gradients mirror known functional findings in the literature. It is hoped that this work will serve as a reminder of the caveat that Brodmann stressed in his landmark work of 1909. Although cortical regions differ from one another, these differences are not as distinct as the reading of modern neuroscience literature may lead one to believe. In the midst of an increasing number of studies attempting to ‘hard parcellate’ the brain, we must remember that the true underlying structure of our data may often be graded.

## Figures and Tables

**Fig. S1:**
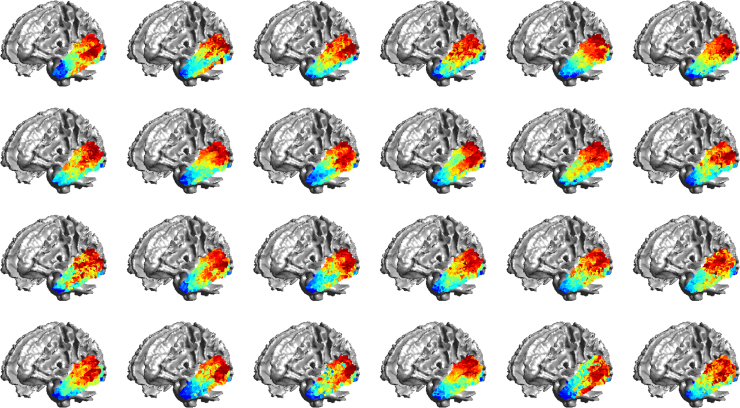
Individual level graded parcellation results of the left temporal lobe projected onto the brain for each of the 24 participants. See Fig. 3 of main article for group level results and explanation of colour coding.

**Fig. S2:**
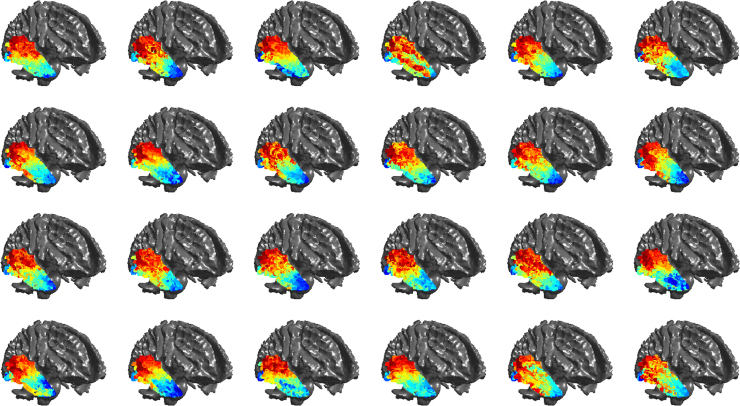
Individual level graded parcellation results of the right temporal lobe projected onto the brain for each of the 24 participants. See Fig. 3 of main article for group level results and explanation of colour coding.
